# Frontal aslant tract in the non-dominant hemisphere: A systematic review of anatomy, functions, and surgical applications

**DOI:** 10.3389/fnana.2022.1025866

**Published:** 2022-11-14

**Authors:** Clémentine Gallet, Anne Clavreul, Florian Bernard, Philippe Menei, Jean-Michel Lemée

**Affiliations:** ^1^Département de Neurochirurgie, CHU d’Angers, Angers, France; ^2^Université d’Angers, Inserm UMR 1307, CNRS UMR 6075, Nantes Université, CRCI^2^NA, Angers, France; ^3^Laboratoire d’Anatomie, Faculté de Médecine d’Angers, Angers, France

**Keywords:** frontal aslant tract, brain anatomy, brain functions, non-dominant hemisphere, awake craniotomy

## Abstract

Knowledge of both the spatial organization and functions of white-matter fiber tracts is steadily increasing. We report here the anatomy and functions of the frontal aslant tract (FAT) in the non-dominant hemisphere (usually the right hemisphere). Despite the structural symmetry between the right and left FAT, these two tracts seem to display functional asymmetry, with several brain functions in common, but others, such as visuospatial and social cognition, music processing, shifting attention or working memory, more exclusively associated with the right FAT. Further studies are required to determine whether damage to the right FAT causes permanent cognitive impairment. Such studies will constitute the best means of testing whether this tract is a critical pathway that must be taken into account during neurosurgical procedures and the essential tasks to be incorporated into intraoperative monitoring during awake craniotomy.

## Highlights

- The FAT: an anatomically symmetric white-matter tract with functional asymmetry.
- The right FAT: a tract of interest supporting key social and cognitive functions.
- The right FAT: a tract to be considered during surgery and perioperative monitoring.

## Introduction

The cerebral white matter was initially described, in the eighteenth century, as a fibrous structure connecting areas of gray matter. The concept of white matter tracts linking various cortical areas in support of particular functions developed much more recently, in the nineteenth century. With improvements in our knowledge of neuroanatomy and dissection techniques, neuroanatomists progressed from a cortical, localizationist view to a more hodologic, network-based vision of brain function. Meynert described three different types of fiber bundles: projection, commissural and association bundles (Meynert, 1892).

Association bundles form strictly intrahemispheric pathways connecting cortical areas within the same hemisphere (Aralasmak et al., 2006). Meynert described them as having only antero-posterior trajectories, a dogma that was not called into question until more than a century later. A vertical fascicle was first described in 2005, in a diffusion tensor imaging (DTI) study by Catani et al. (2005) that identified a vertical perisylvian bundle, the vertical occipital fascicle (VOF), linking the ventral temporo-occipital regions with the cortex surrounding the transverse occipital sulcus and posterior end of the intraparietal sulcus.

Aron et al. (2007) described the first white-matter pathway connecting the inferior frontal cortex with the medial superior frontal cortex. This pathway was named the frontal aslant tract (FAT) by Catani et al. (2012), due to its oblique direction within the frontal lobe (Aron et al., 2007; Catani et al., 2012). Several studies have characterized the connective properties of the FAT, but disagreements remain concerning its precise cortical projections (Dick et al., 2019; Burkhardt et al., 2021; La Corte et al., 2021). It is widely agreed that the FAT connects the pre-supplementary motor area (pre-SMA) and the supplementary motor area (SMA) of the superior frontal gyrus (SFG) to the inferior frontal gyrus (IFG), especially the pars opercularis and, to a lesser extent, the pars triangularis. It remains unclear whether it targets other cortical structures, such as the ventral premotor cortex, the insula, and the postero-lateral and middle parts (anterior to the pre-SMA) of the SFG (Burkhardt et al., 2021). Many studies have assessed the function of the FAT in the dominant hemisphere (usually the left hemisphere) and have shown this tract to be involved in speech initiation, verbal fluency, lexical and semantic word decision, and stuttering (Dick et al., 2019; Burkhardt et al., 2021; La Corte et al., 2021). As a result, surgeons are now advised to identify and preserve this tract during left frontal lobe surgery, to ensure the maintenance of language functions (Chernoff et al., 2019; Monroy-Sosa et al., 2020).

The anatomy and functionality of the left FAT have been described in detail, but much less is known about the right FAT. We performed a literature review, focusing on studies dealing with the anatomy and functions of the right FAT. We also highlight considerations relating to this tract that should be taken into account during surgery.

## Literature review

**FIGURE 1:**
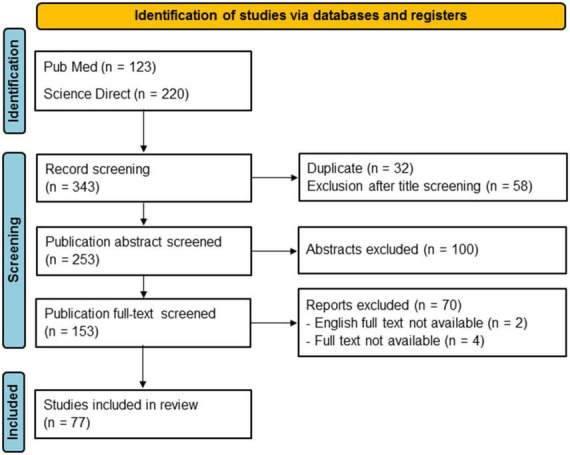
PRISMA flowchart of the systematic review on the right frontal aslant tract (FAT).

This review was conducted according to the Preferred Reporting Items for Systematic Review and Meta-Analyses statement (PRISMA) (Page et al., 2021). PubMed and Science Direct databases were searched with the keywords “frontal AND aslant AND tract.” The search was conducted on February 17, 2022. Records were screened for duplicates and then filtered on the basis of their titles, abstracts, and then the full text, to ensure the selection of relevant studies. We focused, in particular, on anatomical and laboratory papers assessing the orientation of FAT fibers and their cortical origins/terminations through both fiber dissection and DTI studies on humans only. Cortical connections were assessed as described by Destrieux et al. (2017) according to the *Terminologia Anatomica*. We identified 343 articles by database screening, 77 of which were retained after filtering (Figure 1).

## Right frontal aslant tract and anatomy

The first publications on the subject described the FAT as a left-lateralized bilateral tract (Catani et al., 2012; Perry et al., 2015; Rizio and Diaz, 2016; Szmuda et al., 2017; Yeh, 2020). By contrast, recent anatomical studies have described the FAT as a symmetric white-matter tract connecting similar brain areas and with the same diameter and diffusion characteristics on both sides of the brain (Serra et al., 2017; Baker et al., 2018; Briggs et al., 2019, 2020; Keser et al., 2020). The question of white-matter fascicle symmetry between the two hemispheres remains a matter for debate. This issue has also been discussed for several other white-matter bundles, including the arcuate fasciculus (AF). Like the FAT, the AF was initially considered to be an asymmetric white fascicle, but this view was overturned by a recent study by Bernard et al. (2020).

**FIGURE 2:**
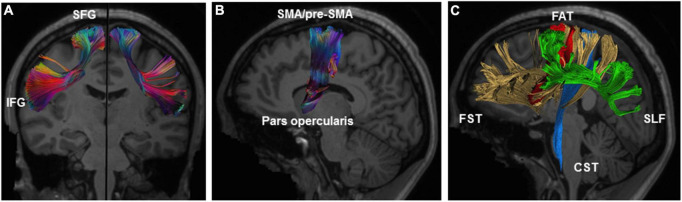
Illustration of the anatomy of the right FAT. **(A)** Coronal view of the left and right FAT with description of connected gyri. **(B)** Sagittal view of the right FAT showing connected brain areas. **(C)** Relationship of FAT (red) to the surrounding white-matter tracts including the SLF (green), FST (yellow), and CST (blue). CST, corticospinal tract; FAT, frontal aslant tract; FST, frontostriatal tract; IFG, inferior frontal gyrus; SFG, superior frontal gyrus; SLF, superior longitudinal fasciculus. Illustration of the frontal aslant tracts based on data freely available from the Human Connectome project and constructed with DSI studio software (https://db.humanconnectome.org; http://dsi-studio.labsolver.org).

The FAT is known to connect the pars opercularis and the pre-SMA/SMA complex, but it has been suggested that other cortical areas, such as the pars triangularis of the IFG, the insula and the ventral premotor cortex, are connected *via* this tract (Bozkurt et al., 2016; Szmuda et al., 2017; Briggs et al., 2018, 2019; Figures 2A,B). The FAT is medial and orthogonal to the superior longitudinal fasciculus (SLF), and anterior to the frontostriatal tract (FST), following a parallel trajectory (Bozkurt et al., 2016; Figure 2C).

One research group has described a bilateral extended FAT (exFAT) with anterior cortical projections in the SFG extending beyond the anterior limit of the pre-SMA (Varriano et al., 2018, 2020; Pascual-Diaz et al., 2020). This group performed a machine learning-validated laterality study with 3T and 7T imaging data from the human connectome project (HCP) dataset, as a means of detecting structural differences between the hemispheres along the exFAT. They found statistically significant differences between left and right fractional anisotropy (FA) values in the central and inferior regions of the exFAT, with a left-lateralization pattern of FA values (Pascual-Diaz et al., 2020). They also suggested that various other segments might be related to different cognitive functions, as described below (Varriano et al., 2018, 2020). Baker et al. (2018) also described a crossed FAT in post-mortem fiber dissection and tractography studies, with fibers arising from SMA splitting into two tracts, one leading to the IFG and striatum and the other to the SMA of the contralateral hemisphere via the corpus callosum. These findings suggested the possibility of interconnection between the FATs of the two hemispheres.

## Right frontal aslant tract and functions

**FIGURE 3:**
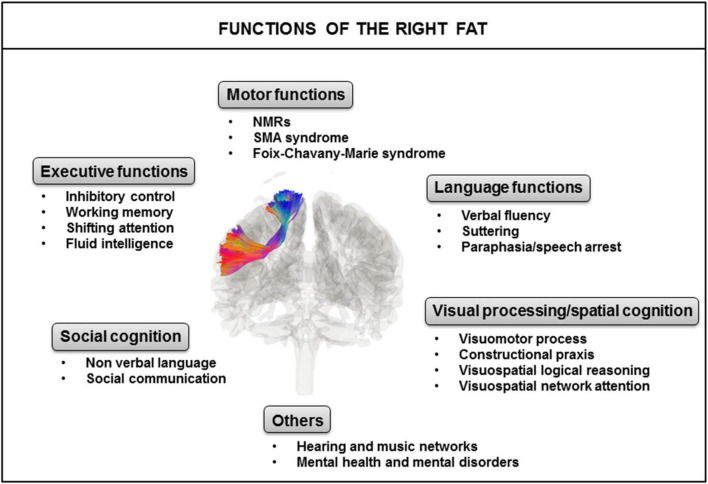
Identified major functions of the right FAT. FAT, frontal aslant tract; NMRs, negative motor responses; SMA, supplementary motor area.

The left and right FAT display structural symmetry, but apparent functional asymmetry with several brain functions in common, but others more exclusively associated with the right FAT or the left FAT. Here, we focus on the global functions identified for the right FAT (Figure 3).

### Motor functions

Several studies have shown both the left and right FAT to be involved in motor functions. For example, according to peroperative observations, FAT and other white-matter pathways may be involved in negative motor responses (NMRs) in the frontal lobe (Kinoshita et al., 2015; Rech et al., 2019; Yokoyama et al., 2020). NMRs are defined as the complete inhibition of movement without the loss of muscle tone or consciousness; they can be elicited by direct electrostimulation of the brain. It has also been suggested that the FAT is an anatomical substrate for SMA syndrome (Pinson et al., 2022). This syndrome is a well-known phenomenon that occurs after medial prefrontal lobe surgery and is characterized by hemiparesis and mutism. It has been argued that postoperative SMA syndrome is transient, but Briggs et al. (2021) found that, in the absence of FAT preservation, 13% of patients presented permanent deficits. The crossed FAT is considered to be the anatomical substrate for recovery from SMA syndrome (Baker et al., 2018). Martino et al. (2012) showed that the resection of connections between the FAT, the AF, and the right pars opercularis of the IFG could cause Foix-Chavany-Marie syndrome, a rare type of suprabulbar palsy characterized by an automatic-voluntary dissociation of the orofacial musculature.

### Language functions

A number of studies have shown that the bilateral FAT plays a role in performance in verbal fluency tasks (Neef et al., 2018; Blecher et al., 2019; Cochereau et al., 2020; Keser et al., 2020; Yablonski et al., 2021; Obayashi, 2022). However, Li et al. (2017) showed that only right FAT volume was positively correlated with scores for semantic fluency in stroke patients. This association was also observed in children with minimally verbal autism spectrum disorder (ASD) (Chenausky et al., 2017). FAT integrity has been found to be affected in stuttering, but it remains unclear whether left or right lateralization of the FAT underlies this speech disorder (Dick et al., 2019; Burkhardt et al., 2021; La Corte et al., 2021). Kronfeld-Duenias et al. (2016) showed, by tractography, that stuttering adults had a lower degree of integrity of both the right and left FAT than controls. Neef et al. (2018) compared tract-based spatial statistics (TBSS), DTI-based probabilistic fiber tracking and functional MRI and showed that the severity of stuttering was associated with the strength of white-matter connections in hyperactive right frontal regions of the brain. In particular, white-matter integrity was compromised in the bilateral SLF and right FAT in stuttering adults (Neef et al., 2018). By contrast, Misaghi et al. (2018) found that the integrity of the right FAT was greater in stuttering children than in controls. Intraoperative electrical stimulation of the right FAT has been reported to induce paraphasia and speech arrest (Rutten et al., 2021). In addition, cathodal transcranial direct current stimulation between the pars opercularis and the pars triangularis of the IFG, an area containing the inferior cortical projects of the FAT, has been reported to increase speed in the picture-naming task (Rosso et al., 2014). Zhong et al. (2022) recently showed that left FAT damage was associated with speech apraxia, an association never previously observed. Further studies are required to evaluate the role of the right FAT in this speech disorder.

### Visual processing and spatial cognition

The role of the FAT in visuomotor processes was highlighted in healthy individuals, in whom a significant correlation between the microstructural characteristics of the bilateral FAT and movement kinematics was observed (Budisavljevic et al., 2017). A higher microstructural organization of the bilateral FAT is associated with lower acceleration and deceleration amplitudes for reach and reach-to-grasp movements, i.e., more efficient visuomotor processing, leading to smoother movement trajectories (Budisavljevic et al., 2017). Serra et al. (2017) found significant positive correlations between the mean FA of the right FAT and the results of tests assessing constructional praxis and visuospatial logical reasoning in patients with Alzheimer’s disease. Constructional apraxia is an acquired deficit of the ability to reproduce spatial relationships occurring in the absence of motor impairments. Takamura et al. (2021) evaluated aspects of attention deficit and neglect symptoms in 174 patients with right hemisphere damage as a function of behavioral inattention test score. Voxel-based lesion symptom mapping revealed many disconnected fasciculi related to visuospatial attention network disorders exclusively in the right hemisphere and commissural fibers, including the FAT.

### Executive functions

The role of the right FAT in executive functions has been highlighted in several studies. Executive functions (including inhibition, working memory, planning, monitoring) are often linked to the frontal lobe, and their impairment probably has significant negative implications for the individual’s social and professional life (Mischel et al., 2011; Moffitt et al., 2011; Duffau and Mandonnet, 2013). Several studies have explored the areas connected by the right FAT, including, in particular, the pre-SMA, SMA and IFG; these areas have been shown to play a role in inhibitory control, conflict monitoring, and working memory (Verbruggen and Logan, 2008; Aron et al., 2014; Erika-Florence et al., 2014; Motomura et al., 2018; Varriano et al., 2018; Dick et al., 2019). These three functions are thought to be involved in top-down executive control (Landers et al., 2021). Rutten et al. (2021) showed that electrical stimulation of the right FAT in a patient with a right frontal low-grade glioma disrupted inhibitory functions monitored by the Stroop task, and working memory assessed with the digit span backward test. Conversely, Puglisi et al. (2019) found no association between right FAT disconnection and postoperative deficits in the Stroop task in patients with frontal right hemisphere glioma. Despite this discrepancy, Varriano et al. (2020) assessed the correlation between tractography-informed voxel-based morphometry analyses of fiber density along the bilateral exFAT and scores for working memory tasks in 35 healthy subjects. They detected the presence of a distinct cluster related to working memory performance corresponding to a novel right anterior FAT component. Landers et al. (2021) found that the right FAT was involved in shifting attention, a top-down executive control process, and letter fluency in patients with frontal brain tumors. No such involvement was observed for the left FAT. Garic et al. (2019) also reported a relationship between the right FAT and attention. By tracking the FAT by diffusion-weighted magnetic resonance imaging (DW-MRI) in 129 typically developing children, they showed that a reduced right laterality of the tract was associated with more severe executive dysfunction, predictive of a greater likelihood of attention problems. Chen et al. (2020) found a significant association between fluid intelligence and bilateral FAT integrity within a multiple-demand system across adult lifespan. Fluid intelligence is the innate ability of an individual to respond to complex and unexpected situations. It is related to general cognition, including logical reasoning, working memory, and decision-making.

### Social cognition

The right FAT may be involved in social cognition and non-verbal language, particularly in theory of mind (ToM), which is the ability to explain and predict other people’s communicative and non-communicative behavior through the attribution of independent mental states (Premack and Woodruff, 1978; Baron-Cohen et al., 1985). A fMRI study on healthy patients was the first to suggest that the bilateral IFG containing the inferior cortical projections of the FAT was involved in ToM and that the right IFG was involved specifically in extralinguistic modalities, such as emotional and non-verbal communication (Tettamanti et al., 2017). This finding was confirmed by Dominguez at al., who showed that disconnection of the right FAT in stroke patients was associated with poor performance in the read-the-mind-in-the-eyes test (RMET), in which the subject is asked to deduce a person’s emotional state based on a picture of their eyes (Domínguez et al., 2019). An association between the integrity of the FAT and ASD, a neurodevelopmental disorder in which one of the main symptoms is a deficit of social communication, has also been highlighted (Chien et al., 2017; Lo et al., 2017, 2019). Lo et al. (2017) showed that there was a lower level of microstructural integrity of the bilateral FAT in boys with ASD than in typically developing boys and that this decrease in integrity was associated with the severity of social interaction deficits. Moreover, they demonstrated that FAT integrity was altered not only in individuals with ASD, but also in their unaffected siblings (Lo et al., 2019). A decrease in bilateral FAT integrity in unaffected siblings of subjects with ASD was also observed by Chien et al. (2017).

### Hearing and music

Several cortical regions within the frontal and temporal lobes have been shown to be involved in the auditory network. Lima et al. (2016) suggested that the SMA and pre-SMA facilitate spontaneous motor responses to sound, and support a flexible engagement of sensorimotor processes underlying imagery and guiding auditory perception. In terms of the lateralization of activity, many studies have reported bilateral SMA and pre-SMA involvement in auditory processing, but further studies are required to address this question (Lima et al., 2016). The cortical regions of the auditory network are linked principally via the AF, FAT and subcortical U-shaped fibers, suggesting a possible involvement of these tracts in auditory processes (Sughrue, 2019; Kuiper et al., 2020). The brain network involved in the processing of music and singing is also highly complex (Särkämö and Sihvonen, 2018). Musical creativity involves three main non-primary motor regions of the frontal lobe: the premotor cortex, SMA/pre-SMA, and posterior IFG (Bashwiner et al., 2020). The FAT, by connecting the SMA and pre-SMA with the IFG, may play a role in musical ability. Studies on amusia, a neurological disorder characterized principally by an inability to perceive fine-grained pitch changes, have shown that severe and persistent amusia after a stroke is associated with damage to and the degeneration of multiple white-matter pathways in the right hemisphere, including the FAT (Sihvonen et al., 2017; Särkämö and Sihvonen, 2018).

### Mental health and mental disorders

The role of the right FAT has been highlighted in some cases of mental health and mental disorders, such as internet gaming disorder (IGD), anosognosia for hemiplegia (AHP), and depressive symptoms. IGD has been defined as a chronic recurrent disorder characterized by compulsive game-seeking, uncontrolled game-playing, and a decision to play despite negative consequences (van Rooij et al., 2017). Zheng et al. (2019) found significant hyperactivity of the right IFG containing the inferior cortical projections of the FAT in IGD patients during risky decision-making tasks. Monai et al. (2020) observed a significant disconnection of the right temporoparietal junction, right insula, right lateral and medial prefrontal cortex in patients with AHP. These associative cortical regions are connected by several white matter tracts, including the superior FAT, AF and longitudinal fasciculus III, fronto-insular, and frontal inferior longitudinal tracts, suggesting that the integrity of these tracts may be affected in AHP. Strain et al. (2013) showed a significant association between the integrity of white-matter tracts, including the right FAT, and the presence of depressive symptoms, in retired National Football League athletes with a history of concussive or subconcussive impacts.

## Right frontal aslant tract and surgical applications

The non-dominant hemisphere was long considered a “minor” hemisphere for which it was not worthwhile taking additional surgical precautions, such as preoperative tractography or surgery in awake craniotomy conditions, such precautions being reserved for the noble “major” hemisphere (Vilasboas et al., 2017). This paradigm is now starting to shift, and more consideration is being accorded to the non-dominant hemisphere, since several reports indicating that quality-of-life impairment after surgery was similar for the left and right hemispheres (Palese et al., 2008; Drewes et al., 2016; Mattavelli et al., 2019). A major role for the non-dominant hemisphere is now recognized in multiple functions, including executive functions, and visuospatial and social cognition (Menei et al., 2017; Bernard et al., 2018b; Lemée et al., 2018). Deficits of these functions after surgery for non-dominant hemisphere lesions are to occur but have been little studied, despite the significant effects they can have on the patient’s social relationships and quality of life.

With increases in our knowledge of the functional anatomy of the dominant and non-dominant hemispheres, new surgical methods have been developed to preserve the functional white-matter pathways of these hemispheres, including the FAT. For example, several authors have called into question the use of Kocher’s point as the standard route of access to the frontal horn of the lateral ventricle, because the FAT is crossed before it penetrates the ventricle. They proposed a new route of access to the ventricle, with a superior frontal sulcus parafascicular point of entry to the frontal horn named the Kassam-Monroy entry point, located 3.5 cm anterior to the coronal suture and 2.3 cm lateral to the superior sagittal sinus. This ventricular access point has been reported to spare most of the white-matter tracts in the frontal lobe, with the exception of the forceps minor, with an anterior course to the FAT (Kassam et al., 2020).

**FIGURE 4:**
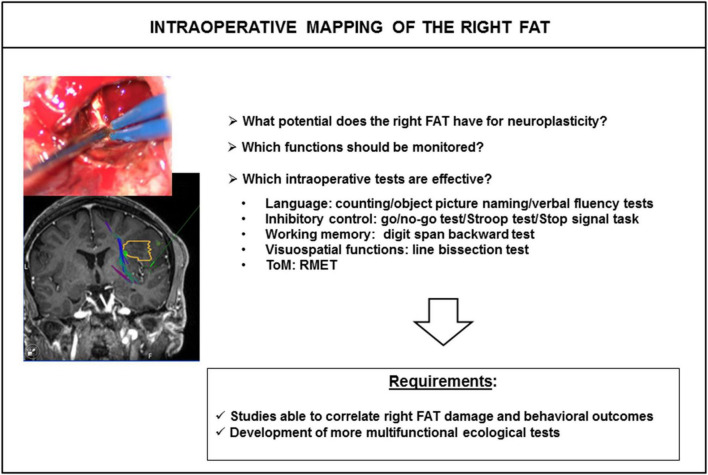
Intraoperative mapping of the right FAT. The answers to several questions are required for effective intraoperative mapping of the right FAT. FAT, frontal aslant tract; ToM, theory of mind; RMET, read-the-mind-in-the eyes test.

Awake craniotomy is also an alternative surgical approach in which different tasks can be tested during surgery, to map and preserve cortical areas and white-matter tracts. It has been shown that white-matter tracts are refractory to functional compensation, particularly for association and projection tracts (Ius et al., 2011; Herbet and Duffau, 2020). Lesions of these tracts lead to permanent deficits. Simply naming or counting tasks have generally been used for the FAT during awake surgery, with the goal of preserving speech initiation and control (Burkhardt et al., 2021). However, few attempts to assess other cognitive functions associated with the FAT, such as executive functions, visuospatial and social cognitions, have been reported (Figure 4). One of the main reasons for the lack of mapping of these functions is the difficulty adapting classic bedside neuropsychological tasks to awake surgery conditions. In particular, the patient must give an unambiguous answer within 5 s, the maximum duration of direct electrical stimulation. We began to address this problem a few years ago, by exploring the feasibility of immersing patients in virtual reality with a virtual reality headset (VRH) during awake brain surgery, as a means of mapping complex cognitive functions (Mazerand et al., 2017; Bernard et al., 2018a,b; Lemée et al., 2018; Delion et al., 2020; Casanova et al., 2021). Various VR experiences were tested, including the picture-naming task, DO 80 and a social VR application vTime^®^, simulating virtual social interactions with an avatar piloted by the neuropsychologist, who also wore a VRH (Bernard et al., 2018a; Delion et al., 2020; Casanova et al., 2021). We observed no VRH-induced intraoperative seizures, or VR sickness (Delion et al., 2020; Casanova et al., 2021).

We recently developed an interactive VR application for the simultaneous analysis of visuospatial cognition and non-verbal language (Casanova et al., 2021). A prospective study (ClinicalTrials.gov NCT04288505) is now underway to determine the specificity and sensitivity of this new ecological VR task. Before the introduction into routine practice of explorations of visuospatial and social functions during awake craniotomy in patients undergoing resections of the FAT or areas close to the FAT, studies are required to confirm that the right FAT is a “long-term eloquent area,” to avoid interference with the onco-functional balance (i.e., the best trade-off between the extent of resection and the preservation of functions) (Mandonnet et al., 2020; Herbet, 2021). The data obtained to date suggest that the FAT is associated with a certain degree of neuroplastic compensation. As indicated above, the SMA syndrome associated with FAT lesions is mostly transient, with spontaneous improvement (Baker et al., 2018; Oda et al., 2018; Briggs et al., 2020, 2021). Herbet and Duffau (2020) generated a probabilistic atlas of functional plasticity based on both anatomic magnetic resonance imaging results and intraoperative mapping data for 231 patients who had undergone surgery for diffuse, low-grade glioma. They suggested that the motor (posterior) segment of the FAT was less likely to undergo functional compensation. Young et al. (2020) reported a case of a patient with a left lower-grade diffuse glioma invading the dominant FAT that was removed during awake craniotomy. The patient displayed immediate postoperative-state language deficits, but quickly recovered, and the patient was neurologically intact at discharge from hospital, a few days after surgery. This suggests that patients can recover after FAT injury, but further studies are required to determine which functions can recover, given the multifunctional properties of this tract, and to determine the mechanisms underlying this recovery: redundancy in the function of the tract, recruitment of other brain regions via other white-matter pathways, or the plasticity of diffuse cortical neural networks. The identification of functions that cannot be recovered after FAT damage will make it possible to find an optimal trade-off between the number of tasks incorporated into the intraoperative battery and the limited amount of time available during awake surgery (about 2 h, corresponding to the time point at which patients become tired, rendering monitoring unreliable) (Mandonnet et al., 2020).

## Conclusion

Frontal aslant tract is a complex tract and further studies, such as connectomic analyses, will be required to characterize its connections and segmentation in more detail. Moreover, as highlighted by Burkhardt et al. (2021) analyses of the interindividual variability of FAT projections are still lacking. The right and left FAT are structurally symmetric, but functionally asymmetric. Several brain functions involve the bilateral FAT, but others, such as visuospatial and social cognition, music processing, shifting attention, or working memory, are more exclusively associated with the right FAT. More studies are needed to determine whether damage to the right FAT causes permanent cognitive impairment. Such studies are the best way to determine whether this tract is a critical pathway that must be taken into account during neurosurgical procedures and the essential tasks that should be incorporated into intraoperative monitoring during awake craniotomy.

## Author contributions

CG and J-ML wrote the first draft of the review. AC and J-ML performed critical revision of the manuscript. All authors commented on intermediate versions of the review, read, and approved the final review.
